# Accuracy of implant placement with computer-aided static, dynamic, and robot-assisted surgery: a systematic review and meta-analysis of clinical trials

**DOI:** 10.1186/s12903-024-04033-y

**Published:** 2024-03-21

**Authors:** Angkoon Khaohoen, Warit Powcharoen, Tanapon Sornsuwan, Pisaisit Chaijareenont, Chaiy Rungsiyakull, Pimduen Rungsiyakull

**Affiliations:** 1 Department of Prosthodontics, Faculty of Dentistry, Chiang Mai University, Chiang Mai, 50200 Thailand; 2 Department of Oral and Maxillofacial Surgery, Faculty of Dentistry, Chiang Mai University, Chiang Mai, 50200 Thailand; 3 Department of Restorative Dentistry, Faculty of Dentistry, Naresuan University, Phitsanulok, 65000 Thailand; 4 Department of Mechanical Engineering, Faculty of Engineering, Chiang Mai University, Chiang Mai, 50200 Thailand

**Keywords:** Robotic guide surgery, Image-guided surgery, Computer-assisted implantation, Surgical navigation, Dental implants, Systematic review

## Abstract

This systematic review explores the accuracy of computerized guided implant placement including computer-aided static, dynamic, and robot-assisted surgery. An electronic search up to February 28, 2023, was conducted using the PubMed, Embase, and Scopus databases using the search terms “surgery”, “computer-assisted”, “dynamic computer-assisted”, “robotic surgical procedures”, and “dental implants”. The outcome variables were discrepancies including the implant’s 3D-coronal, -apical and -angular deviations. Articles were selectively retrieved according to the inclusion and exclusion criteria, and the data were quantitatively meta-analysed to verify the study outcomes. Sixty-seven articles were finally identified and included for analysis. The accuracy comparison revealed an overall mean deviation at the entry point of 1.11 mm (95% CI: 1.02–1.19), and 1.40 mm (95% CI: 1.31–1.49) at the apex, and the angulation was 3.51˚ (95% CI: 3.27–3.75). Amongst computerized guided implant placements, the robotic system tended to show the lowest deviation (0.81 mm in coronal deviation, 0.77 mm in apical deviation, and 1.71˚ in angular deviation). No significant differences were found between the arch type and flap operation in cases of dynamic navigation. The fully-guided protocol demonstrated a significantly higher level of accuracy compared to the pilot-guided protocol, but did not show any significant difference when compared to the partially guided protocol. The use of computerized technology clinically affirms that operators can accurately place implants in three directions. Several studies agree that a fully guided protocol is the gold standard in clinical practice.

## Background

Currently, implants to replace missing teeth play a significant role in dental treatment because of their exceptional survival rate in restoring both partial and complete edentulism [1, 2]. To improve the long-term success rate of final implant prostheses, prosthetically-driven implant placement with computerized implant technology is the most crucial factor in overcoming a placement’s early or late failure [3, 4].

Cone-beam computed tomography (CBCT) is used in implant planning to generate multiplanar and three-dimensional pictures that enhance the clinical outcome. By incorporating CBCT into implant planning, clinicians can evaluate the condition of the alveolar bone, which is directly related to the success rate of implants, in order to predict primary stability [5]. This information can assist the surgeon in choosing suitable dental implant and surgical techniques prior to careful placement. In addition, CBCT combined with digital scanning images can be transferred to specific software and utilized to anticipate virtual crowns before the intraoperative procedure [6].

Recently, computerized guided implant placement has been widely used to assist operators in reducing the unexpectedly deviated position and unfortunate results after osteotomy. This system can be divided into three subgroups: static, dynamic computer-aided, and robot-assisted implant surgery [7]. Static computer-aided implant surgery (sCAIS) systems are digitally programmed fabricated surgical templates designed to represent the final implant’s position [8]. Dynamic CAIS (dCAIS) provides a real-time procedure related to the dental drill using optical or mechanical tracking technology without a template guide co-operated through a nearby monitor [9]. Nevertheless, d-CAIS is devoid of physical guidance and has an associative learning curve [10]. Simultaneously, robotic CAIS (rCAIS) combines the benefits of avoiding the physical constraints of s-CAIS, the instantaneous feedback of d-CAIS, and the accurate control achieved by robotic arms [11]. Robot-assisted implant systems have been developed to improve precision and accuracy, lessen human-based errors, and eliminate the use of static guides [12]. Yomi [11] is the first robotic implant surgical system approved by the Food and Drug Administration (FDA). It is known as a semi-active robot assistance system since the surgeon can manually conduct the implant osteotomy through a robotic arm. Recently, China’s National Medical Product Administration authorized Remebot, an autonomous robot-assisted surgery system. In addition to being a semi-active device, it is also a task-autonomous robotic system that can drill and place the implant autonomously in the planned position [13].

Even though digital implant technology assists in reducing the errors cause by operator- or patient-related factors, the accumulative errors in computer-guided systems still exist as the result of preoperative or intraoperative procedures or postoperative evaluation [9], as shown in Table 1. The flaws of navigation systems have been clinically reported. For instance, a common weak point in a static surgical guide is the manufacturing error, which can hamper the proper positioning and stability of the guide during the operation [8, 14]. The occurrence of errors in guide systems can be influenced by several factors, including the quality of the acquired image and the expertise and understanding of the system's operator. Dynamic navigation, on the contrary, can guide the osteotomy and implant without template guidance. Some authors claim that the learning curve is influenced by the operator’s personal confidence with the computer and the 3D system [15, 16]. However, this system appears to provide more advantages for novice professionals by minimizing their mistakes. Put simply, it has the potential to enhance the proficiency of novice surgeons. Although robot-assisted dental surgery provides haptic and visual guidance during implant surgery [11], the sum of errors can derive from the complexity of multiple Digital Imaging and Communications in Medicine (DICOM) files and the instability of polymethyl methacrylate (PMMA) guide fixation. The drawback to using multiple superimposition phases involves the utilization of various DICOM files or CBCT scans, including the preoperative and intraoperative scans. Each instance of superimposition has the potential to lead to mistakes. Hence, it is imperative to meticulously verify every step of the merging process for correctness. Furthermore, the attachment of PMMA guides presents a challenging scenario. Unstable occlusal splint guidance might lead to inaccurate implant placement. In instances such as those involving movable teeth, large crowns and bridges, or many dental implants, an edentulous splint can be utilized instead of a dentate splint. There are individuals who have an allergy to PMMA. Two potential options for splinting are the new dentate splint or the clamp splint [7, 17]. The advantages and disadvantages of robotic systems compared to static and dynamic systems are summarized in Table 2.

**Table 1:** The cumulative errors* of static, dynamic, and robotic systems

**Error Procedure**	**Errors sources**	**Static system **[18–24]	**Dynamic system **[15, 24–27]	**Robotic system **[11, 13, 27, 28]
**Preoperative** Patient to software	*Data registration* Impression taking (STL files) and Imaging (CBCT)	- Impression materials,techniques, and scanner machine - CT machine and scanning method	- CT machine and scanning method - Location and quantity of fiducial markers - Tracking system	- CT machine and scanning method - Location and quantity of fiducial markers - Tracking system
	*Superposition* Merging of CBCT-STL or CBCT-CBCT file images	- Implant software planning - Remaining teeth	- Implant software planning	- Implant software planning
	*Guide manufacturing*	- Type of guide machine and material	No	No
**Intraoperative** software to patient		- Positioning and stability of the guide - Mucosal thickness - Guide support (tooth-, mucosa-, or bone) - Template fracture or metal sleeve disintegration from the guide - Surgical guide protocol - Arch and site of operation - Flap operation	- Attachment of guide - Calibrate the relationships of the hand instrument and the orientation of the head of the patient with CBCT image - Surgeon’s experience	- Attachment of guide - Calibrate the relationships of the robotic arm and the orientation of the head of the patient with CBCT
**Postoperative** Assessment tool		Postoperative implant placement position technique		

^***^ Cumulative error refers to the discrepancy between the planned and actually placement of the implant in terms of its 3D position and angulation during the entire operation

**Table 2:** Advantages and disadvantages of robotic systems compared to static and dynamic systems. [7, 8, 11, 14–17]

	**Static system**	**Dynamic system**	**Robotic system**
Advantage	- Physical guidance for implantation	- Real-time visual feedback to surgeons	- Combining the flexibility and visuality of dynamic navigation with the physical constraints of static guides results in high accuracy and dependability
Disadvantage	- Cannot be adjusted and may shift or fracture intraoperatively - Utilization is restricted by undesirable water cooling, poor visibility, and the patient’s small mouth opening, particularly in posterior sites	- Marked learning curve - Human errors, such as hand tremors, and the lack of experience of the surgeons	- Intrinsic errors from CBCT data acquisition, calibration, registration, and the error of the robotic arm - Attachment of PMMA guides - Time consuming - High purchase cost

Several previous systematic reviews have summarized the accuracy of static and dynamic computer-assisted systems and the factors relating to their accuracy. The findings indicate that the utilization of d-CAIS enhanced the accuracy of implant placement in comparison to the freehand technique, and also modestly reduced the angular deviation when compared to s-CAIS [10]. In addition, it provided a safety range that is considered appropriate for clinical use [9]. Nevertheless, there have been inadequate studies investigating the use of robot-assisted implant systems in clinical trials, and no systematic analysis has been conducted to compare the static, dynamic, and robot-assisted systems.

Regarding static systems, Tahmaseb, *et.al*. [29] found that partial edentulism is often more favorable than complete edentulism, except in angular deviation. Zhou and colleagues [20] concluded that a fully guided protocol, which involves employing a fixation screw and a flapless technique, showed the most remarkable accuracy. Furthermore, they stated that there was a statistically significant difference in angular deviation, favoring the mandibular arch over the maxillary arch. In cases of dynamic systems, Wei and colleagues [9] evaluated the accuracy of d-CAIS and identified the factors that influence its accuracy. However, the impact of significant clinical factors on the accuracy of the navigation system is obsolete, and some clinical aspects have not been assessed, such as the lack of comparisons regarding the surgical protocol in the static system or the arch and flap techniques in the dynamic system.

The key difference in this systematic review, in comparison to others, is the separate evaluation of robot guided surgery and its comparison to other subgroups. Therefore, this systematic review aims to explore the clinical accuracy of computerized guided surgery for dental implant placement based on a virtual plan and later aims to identify the relevant factors affecting its accuracy.

## Methods

Our review was enrolled in the International Prospective Register of Systematic Reviews (PROSPERO); the registered protocol number is CRD42022332900. This systematic review performed documentation management following the Preferred Reporting Item for Systematic Reviews and Meta-analysis (PRISMA) guidelines.

### PICO question

Based on the PRISMA guidelines, our systematic review illustrated the accuracy of implant placement using computer-aided static, dynamic, and robot-assisted surgery. The cornerstone question was: “What is the accuracy of a computer-guided implant system in relation to the preoperative plan?”.

### Search strategy

The formulation of a research question following the PICO methodology was used to define the scope. An electronic and manual search of PubMed, Embase, and Scopus revealed limited sources with the identified relevant keywords included in the title, abstract, and subject terms.

An initial search was performed with the terms, as shown in Table 3 [9, 10]. Papers published in the last thirteen years (between January 1, 2010 and February 28, 2023) in the English language and indexed in the aforementioned databases were searched.

**Table 3:** Search strategy according to three databases (PubMed, Scopus, Embase)

PICO question		Search strategy [9, 10]		
		PubMed	Scopus	Embase
P	Patients treated with dental implants placement,	("Surgery, Computer-Assisted"[MeSH] OR "Robotic surgical procedures"[MeSH] OR "Computer-assisted surgery" OR "navigation system" OR "navigation systems" OR "dynamic computer-aided" OR "dynamic computer guided" OR "dynamic computer assisted" OR robot* OR Yomi) AND ("Dental Implants" OR "tooth implant" OR "single tooth implant" OR "dental implants" OR "dental implant" OR implantology)	TITLE-ABS-KEY ("Surgery, Computer-Assisted" OR "Robotic surgical procedures" OR "Computer-assisted surgery" OR "navigation system" OR "navigation systems" OR "dynamic computer-aided" OR "dynamic computer guided" OR "dynamic computer assisted" OR robot* OR yomi) AND TITLE-ABS-KEY ( "Dental Implants" OR "tooth implant" OR "single tooth implant" OR "dental implants" OR "dental implant" OR implantology)	('surgery, computer-assisted'/exp OR 'surgery, computer-assisted' OR 'robotic surgical procedures'/exp OR 'robotic surgical procedures' OR 'computer-assisted surgery'/exp OR 'computer-assisted surgery' OR 'navigation system'/exp OR 'navigation system' OR 'navigation systems' OR 'dynamic computer-aided' OR 'dynamic computer guided' OR 'dynamic computer assisted' OR robot* OR yomi) AND ('tooth implant'/exp OR 'tooth implant' OR 'single tooth implant'/exp OR 'single tooth implant' OR 'dental implants'/exp OR 'dental implants' OR 'dental implant'/exp OR 'dental implant' OR 'implantology'/exp OR implantology)
I	Computerized-implant dentistry; sCais, dCais and robotic-assisted implant surgery,			
C	Pre- and post-operative implant placement			
O	Accuracy of dental implant placement; three-dimensional coronal, -apical, and -angular directions,			
S	Randomized or non-randomized controlled trials (retrospective and prospective trials) and case studies			

### Study selection

Two reviewers (K.A. and R.P.) screened all abstracts and titles independently. In the event of disagreements or arguments, the discussion was solved by consensus or a third opinion (C.P.). No kappa score was computed. The reasons for the exclusion of the abstracts and titles that were not further included in this review were that (a) they comprised review literature and preclinical studies, (b) they were on unrelated topic and dental implants, or (c) the parameters were unclear. The articles retrieved from the search and the inclusion and exclusion criteria were obtained for data synthesis. For evaluation, the full-text criteria are listed in Table 4.

**Table 4:** Inclusion and exclusion criteria for selected trials

Inclusion criteria	Exclusion criteria
Randomized or nonrandomized controlled trials, prospective, and retrospective clinical studies, case studies or case series	Review articles or expert opions, case studies or case series (less than five implants), and preclinical studies
CT or CBCT scans that were used for computerized planning in s-CAIS, d-CAIS, 3D-augmented reality and robotic-assisted surgery;	Patient with zygomatic implants or mini-implants for orthodontic purposes;
Intraoral scanning and extraoral scanning that were used for merging the data planning;	Studies involving MRI or panoramic approaches for planning or determining the accuracy;
Partially or completely edentulous sites	
Implant site preparation and implant insertion were included;	
Deviation between virtual planned and actual positions were digital measurement such as non-radiographic and radiographic methods;	
Measurement of all studies were the outcome of accuracy and clear description on accuracy measurements including 3D-coronal, 3D-apical, and angular deviation;	
The review wrote in English language and both abstract and full article available	

*CT* Computed tomography, *CBCT* Cone beam computed tomography, *s-CAIS* Static computer-aided implant surgery, *d-CAIS* Dynamic computer-aided implant surgery, *MRI* Magnetic resonance imaging, *3D* 3 dimensional

### Data extraction

**Fig. 1:**
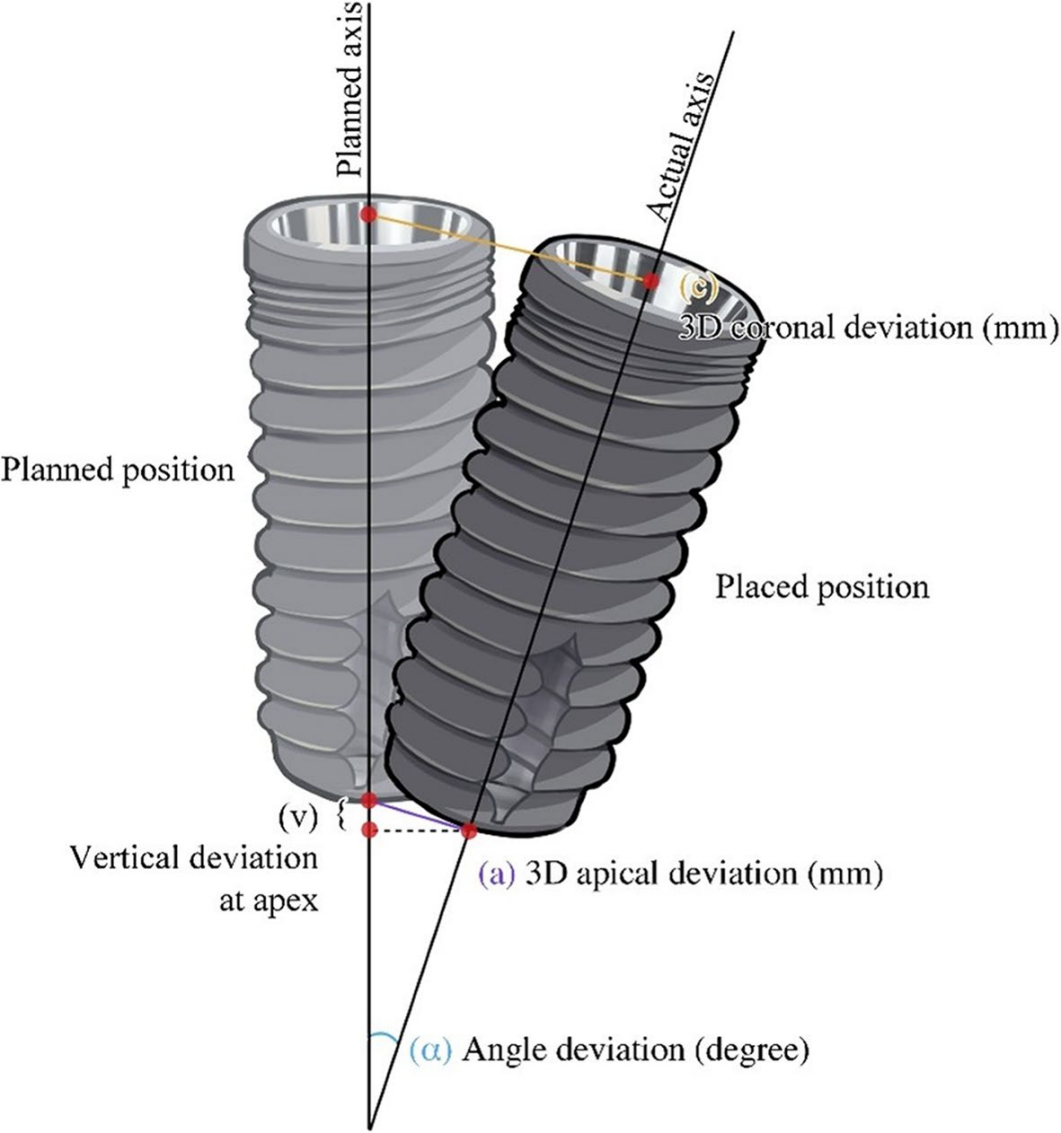
Illustrates the analytic parameters of the accuracy of dental implant navigation systems in this review study. Global angular (α) is the 3D angle between the central axis of the planned and the placed position. Global coronal (c) is the 3D distances between the coronal centers of the planned and actual position. Global apical (a) is the 3D distances between the apical centers of the planned and actual position

After the first author (K.A.) and second author (R.P) independently selected and retrieved articles against the inclusion criteria, the data from the articles were collected and organized, and the extracted data were recorded in an Excel spreadsheet (Microsoft Corporation, Redmond, WA, USA). The data were grouped to analyze the following variables as shown in Tables 5, 6, 7 and 8. Each system was separated into static, dynamic, and robotic groups to evaluate the discrepancies: deviation at the entry point and the apex in a three-dimensional direction and deviation of the axis (Fig. 1). The data on the flap operation (flapless or open flap) and different jaw bones involved in implant placement (maxilla or mandible) in dynamic navigation systems were recorded. Additionally, static systems were analyzed regarding whether the surgical protocol was a pilot or partial or full protocol.

**Table 5:** Detail of the included studies of the static navigation system

Author	Study design	Sample (implant)	Clinical condition		Pre-operative planning		Surgical procedure				Evaluation method
			Arch (implant)	Edentulisn (implant)	Scanning	Software	Flap method (implant)	Guide support (implant)	Protocol		
Assche et al., 2010 [30]	Pros	19	Max (17) Man (2)	Partial	No	Procera®	NR	Tooth	Fully		Radiographic
Arisan et al., 2010 [31]	Retro	279	Max Man	Partial Complete	No	Stent CAD® SimPlant Pro®	Flap-Flapless	Tooth (95) Mucosa (97) Bone (87)	Partial Fully		Radiographic
Cassetta et al., 2011 [32]	Retro	111	Max (68) Man (43)	Partial (17) Complete (94)	No	Simplant®	Flap (18) Flapless (93)	Tooth (8) Bone (18) Mucosa (85)	Fully		Radiographic
Cassetta et al., 2012 [33]	Retro	116	Max (67) Man (49)	Partial (28) Complete (88)	No	Simplant®	Flap (22) Flapless (94)	Tooth (15) Mucosa (88) Bone (13)	NR		Radiographic
Pettersson et al., 2012 [19]	Retro	139	Max (89) Man (50)	Complete	No	Procera®	Flapless	Bone	Fully		Radiographic
Cassetta et al., 2012 [34]	Retro	95	Max	Complete	No	Simplant®	Flapless	Mucosa	NR		Radiographic
D'Haese et al., 2012 [18]	Pro	77	Max (77)	Complete	No	Astra Tech AB®	Flapless	Mucosa	Fully		Radiographic
Cassetta et al., 2013 [35]	Retro	111	NR	Partial Complete	No	Simplant®	Flap-Flapless	Tooth Mucosa Bone	Fully		Radiographic
Cassetta et al., 2013 [36]	Retro	227	Max (135) Man (92)	Partial (45) Complete (182)	NR	Simplant®	Flap (40) Flapless (187)	Tooth Mucosa Bone	Fully		Radiographic
Cassetta et al., 2013 [37]	Retro	129	Max (78) Man (51)	Partial (17) Complete (112)	No	Simplant®	Flap (18) Flapless (111)	Tooth (8) Mucosa (103) Bone (18)	NR	Radiographic	
Van de Wiele et al., 2014 [38]	Pro	75	Max-Man	Complete	No	Simplant®	Flapless	Mucosa	NR	Radiographic	
Testori et al., 2014 [39]	Pro	117	Max-Man	Complete	NR	Simplant®	NR	Mucosa Bone Tooth	NR	Radiographic	
Verhamme et al., 2015 [40]	Pro	150	Max (150)	Complete	No	Procera®	NR	Mucosa	NR	Radiographic	
Alzoubi et al., 2016 [41]	Retro	40	Max-Man	Partial	IOS EOS	Anatomage®	NR	Tooth	Fully	Radiographic	
Cassetta and Bellardini, 2017 [42]	RCT	C: 33 T: 37	Max-Man	Complete	No	3Diagnosys®	Flapless	Mucosa	Fully	Radiographic	
Horwitz et al., 2017 [43]	Case series	18	Max-Man	Partial	NR	SMOP®	Flap	Tooth	Fully	Radiographic	
Albiero et al., 2017 [44]	Case series	60	Max (42) Man (18)	Complete	NR	Simplant®	Flapless	Mucosa	Fully	Radiographic	
Schnutenhaus et al., 2018 [45]	Retro	122	Max (48) Man (74)	Partial	EOS	SMOP®	Flap-Flapless	Tooth	Fully	Non-radiographic	
Schnutenhaus et al., 2018 [46]	Pro	20	Max (10) Man (10)	Partial	EOS	SMOP®	Flap-Flapless	Tooth	Fully	Non-radiographic	
Derksen et al., 2019 [21]	Pro	145	Max (66) Man (79)	Partial	IOS	CoDiagnostix®	Flap (111) Flapless (34)	Tooth	Fully	Non-radiographic	
Albiero et al., 2019 [47]	Retro	114	Max (88) Man (26)	Partial Complete	No	Simplant®	Flapless	Mucosa	Fully	Radiographic	
Schelbert et al., 2019 [48]	Retro	26	Max (11) Man (15)	Partial	IOS	SMOP®	Flap	Tooth Mucosa	Fully	Radiographic	
Zhou et al., 2019 [49]	Pro	74	Max-Man	Partial Complete	IOS	GuideMia®	Flap	Tooth Mucosa	Partial	Radiographic	
Skjerven et al., 2019 [50]	Pro	27	Max (21) Man (6)	Partial	IOS	Implant Studio®	Flap	Tooth	Fully	Non-radiographic	
Monaco et al., 2020 [51]	Retro	120	Max (61) Man (59)	Partial Complete	IOS	CoDiagnostix®	Flap (96) Flapless (24)	Tooth mucosa	Fully	Non-radiographic	
Suksod et al., 2020 [52]	Pro	20	Max (16) Man (4)	Partial	NR	CoDiagnostix®	Flap	Tooth	Partial	Radiographic	
Kiatkroekkrai et al., 2020 [22]	RCT	C: 30 T: 30	Max (40) Man (20)	Partial	IOS EOS	CoDiagnostix®	Flap-Flapless	Tooth	Fully	Radiographic	
Chai et al., 2020 [53]	Pro	44	Max (21) Man (23)	Complete	EOS	Organical® Dental Implant®	Flapless	Mucosa	Fully	Radiographic	
Kivovics et al., 2020 [54]	RCT	C: 22 T: 18	Max-Man	Complete	EOS	CoDiagnostix®	Flap	Mucosa	Partial	Radiographic	
Cassetta et al., 2020 [8]	Pro	56	Max-Man	Partial (23) Complete (33)	NR	3Diagnosys®	NR	Tooth Mucosa	Fully	Radiographic	
Zhang et al., 2021 [55]	Pro	30	NR	Partial	IOS	Implant Studio®	Flap	Tooth	Fully	Radiographic	
Cunha et al., 2021 [56]	Pro	61	Max (36) Man (25)	Complete	No	P3Dental®	Flapless (61)	Mucosa (61)	Fully	Radiographic	
Cho et al., 2021 [57]	Pro	48	Max (39) Man (9)	Partial	IOS	Blus Sky Plan III®	NR	Tooth	Partial	Radiographic	
Gargallo et al., 2022 [58]	Pro	60	Max (8) Man (52)	Partial	IOS	CoDiagnostix®	Flap	Tooth	Fully	Radiographic	
Cristache et al., 2021 [59]	RCT	C: 56 T: 55	Max (46) Man (65)	Partial	IOS EOS	R2gate®	Flapless	Tooth	Fully	Non-radiographic	
Han et al., 2021 [60]	Retro	74	Max (23) Man (33)	Partial Complete	EOS	BenQ AB®	NR	Tooth (28) Mucosa (28)	Partial Fully	Radiographic	
Schnutenhaus et al., 2021 [61]	Pro	20	Max (8) Man (12)	Partial (20)	EOS	SMOP®	Flap	Tooth	Fully	Non-radiographic	
Matsumura et al., 2021 [62]	Retro	188	Max-Man	Partial	NR	CoDiagnostix®	NR	Tooth	Fully Partial	Radiographic	
Souza et al., 2022 [63]	Retro	54	Max-Man	Partial	EOS	CoDiagnostix®	Flap-Flapless	Tooth Mucosa	Fully	Non-radiographic	
Sun et al., 2022 [64]	RCT	C: 15 T: 15	Max (14) Man (16)	Partial	IOS	CoDiagnostix®	NR	Tooth	Partial	Non-radiographic	
Orban et al., 2022 [65]	RCT	C: 20 T: 20	Max	Partial	NR	NR	Flap	Tooth Mucosa	Partial	Non-radiographic	
Ngamprasertkit et al., 2022 [66]	RCT	C: 15 T: 15	Max-Man	Partial	IOS	Planmeca Romexis®	Flap	Tooth	Partial Fully	Radiographic	
Zhu et al., 2022 [67]	Retro	191	Max (91) Man (100)	Complete	No	6D Planning®	Flapless	Mucosa	Partial	Radiographic	

*Abbreviations*: *RCT* Randomized controlled trial, *Pro* Prospective, *Retro* Retrospective, *Max* Maxilla, *Man* Mandible, *NR* No record, *IOS* Intraoral scanner, *EOS* Extraoral scanner, *C* Control group, *T* Test group

**Table 6:** Detail of the included studies of the dynamic navigation system

Author	Study design	Sample (implant)	Clinical condition		Pre-operative planning		Surgical procedure			Evaluation method
			Arch (implant)	Edentulisn (implant)	Scanning	Software	Flap method (implant)	Guide support (implant)	Protocol	
Pellegrino et al., 2019 [16]	Pro	18	Max (9) Man (9)	Partial Complete	No	ImplaNav®	Flap-Flapless	No	NR	Radiographic
Stefanelli et al., 2020 [25]	Retro	136	Max (75) Man (61)	Partial	IOS	Navident®	NR	No	NR	Radiographic
Edelmann et al., 2021 [68]	Pro	C: 10 T: 10	Max (4) Man (16)	Partial	IOS	CoDiagnostix®	Flap (10) Flapless (10)	NR	NR	Radiographic
Wei et al., 2022 [69]	RCT	20	Max (20)	Partial	No	Dcarer®	Flapless (20)	No	NR	Radiographic
Zhang et al., 2022 [7]	Retro	48	Max	Complete	IOS	Dental Implant Navigation®	Flap	No	No	Radiographic
Ma el al., 2022 [70]	Retro	99	Max-Man	Partial	No	Dcarer®	Flap	No	No	Radiographic
Van Hooft et al., 2022 [71]	Case series	23	Max-Man	Partial	IOS	DTX studio®	Flap	No	Fully	Radiographic Nonradiographic

*Abbreviations*: *RCT* Randomized controlled trial, *Pro* Prospective, *Retro* Retrospective, *Max* Maxilla, *Man* Mandible, *NR* No record, *IOS* Intraoral scanner, *C* Control group, *T* Test group

**Table 7:** Detail of the included studies of the robotic navigation system

Author	Study design	Sample (implant)	Clinical condition		Pre-operative planning		Surgical procedure			Evaluation method
			Arch (implant)	Edentulisn (implant)	Scanning	Software	Flap method (implant)	Guide support (implant)	Protocol	
Bolding and Reebye, 2021 [11]	Pro	38	Max (15) Man (23)	Complete	No	Neocis Inc®	Flapless	No	No	Radiographic
Yang et al. et al., 2022 [13]	Case report	6	Max	Complete	No	CoDiagnostix	Flapless	Tooth-mucosa	No	Radiographic

*Abbreviations*: *Pro* Prospective, *Max* Maxilla, *Man* Mandible, *NR* No record

**Table 8:** Detail of the included studies of the static, dynamic and, robotic navigation system

Author	Study design	Sample (implant)	Navigation system	Clinical condition		Pre-operative planning		Surgical procedure			Evaluation method
				Arch (implant)	Edentulisn (implant)	Scanning	Software	Flap method (implant)	Guide support (implant)	Protocol	
Vercruyssen et al., 2014 [72]	RCT	C: 102 T: 209	Freehand (102) Static (209)	Max-Man	Complete	No	Simplant®	Flap Flapless	Bone (102) Mucosa (107)	NR	Radiographic
Shen et al., 2015 [73]	Pro	109	Freehand (52) Static (57)	NR	Partial	NR	Simplant®	NR	NR	NR	Radiographic
Smitkarn et al., 2019 [74]	RCT	C: 30 T: 30	Freehand (30) Static (30)	Max (39) Man (21)	Partial	IOS	CoDiagnostix®	Flap	Tooth	Fully	Radiographic
Schneider et al., 2019 [75]	RCT	C: 16 T: 41	Freehand (16) Static (41)	Max-Man	Partial (57)	No	Simplant® SMOP®	Flap	Tooth	Fully	Non-radiographic
Younes et al., 2018 [76]	RCT	C: 26 T: 45	Freehand (26) Static (45)	Max (71)	Partial (71)	EOS	Simplant®	Flap Flapless	Tooth	Pilot Fully	Radiographic
Varga et al., 2020 [77]	RCT	C: 55 T: 152	Freehand (55) Static (152)	Max (137) Man (70)	Partial	No	SMART Guide®	Flap Flapless	Tooth	Pilot Partial Fully	Radiographic
Tencati and Moy et al., 2019 [78]	Retro	202	Freehand (102) Static (86) Dynamic (14)	Max-Man	Partial	NR	NR	Flap	NR	NR	Radiographic
Block et al., 2017 [15]	Pro	714	Freehand (122) Dynamic (592)	NR	NR	No	X-Nav®	NR	No	Half Fully	Radiographic
Aydemir and Arisan et al., 2020 [26]	RCT	C: 43 T: 43	Freehand (43) Dynamic (43)	Max(86)	Partial	No	Navident®	Flap	No	No	Radiographic
Kaewsiri et al., 2019 [79]	RCT	C: 30 T: 30	Static (30) Dynamic (30)	Max(37) Man(23)	Partial	IOS EOS	CoDiagnostix®	Flap (50) Flapless (10)	Tooth	Fully	Radiographic
Yimarj et al., 2020 [12]	RCT	C: 30 T: 30	Static (30) Dynamic (30)	NR	Partial (60)	EOS	CoDiagnostix®	NR	Tooth	Fully	Radiographic
Nomiyama et al., 2022 [80]	RCT	C: 85 T: 86	Freehand (85) Static (86)	Max	Complete	No	Dental Slice Virtual Navigation®	Flap Flapless	Mucosa	Pilot Fully	Radiographic
Wei el al., 2022 [81]	RCT	C: 12 T: 12	Freehand (12) Dynamic (12)	Max	Partial	IOS	Dcarer®	Flapless	NR	Fully	Radiographic
Feng el al., 2022 [82]	RCT	C: 20 T: 20	Static (20) Dynamic (20)	Max	Partial	IOS	NobelClinician® Dcarer®	Flap Flapless	Tooth No guide	Fully	Radiographic
Jaemsuwan el al., 2022 [83]	Pro	60	Freehand (20) Static (20) Dynamic (20)	Max-Man	Complete	EOS	CoDiagnostix® IRIS 100®	Flap	Bone No guide	Fully	Radiographic

*Abbreviations*: *RCT* Randomized controlled trial, *Pro* Prospective, *Max* Maxilla, *Man* Mandible, *NR* No record, *IOS* Intraoral scanner, *EOS* Extraoral scan, *C* Control group, *T*:Test group

### Quality assessment

The quality of the included RCTs was evaluated using the Cochrane risk of bias (RoB-2) tool [84]. Relying on the descriptions given for each criterion, a scoring of low concern, some concern, or a high risk of bias was assigned. At the same time, the Robins-I was used to evaluate the risk of bias in the non-randomized clinical trials that were included [85]. The rating of each criterion was the same using RCT. Furthermore, four case studies were analyzed using JBI checklist [86].

### Statistical analysis

Statistical analysis was performed using RevMan 5 (Review manager version 5; The Cochrane Collaboration, Copenhagen, Denmark) and Microsoft Excel: Meta-Essentials workbooks for meta-analysis version 1.5 (Suurmond R et al., 2017) [87]. The overall accuracy of computerized guided implant placement and selected influential factors were evaluated. Due to the heterogeneity between the articles, totals were evaluated using random-effects models for continuous variables. Three parameters (coronal, apical, and angular deviations) were analyzed separately. Additionally, forest plots were used to estimate the overall results from the mean and standard deviation (SD) weighted by the size of each group and the means of the meta-analysis had a corresponding 95% confidence interval. The significance level of the tests was 0.05 [88].

The navigation system group is reported in the descriptive analysis. Pairwise meta-analyses were performed to evaluate the factors of flap operation, arch type, and guided surgery protocol wherein the mean differences were evaluated using random-effect models.

### Heterogeneity and publication bias

The Q heterogeneity statistic and corresponding P value for the chi-squared test were analyzed. A P value of the Q statistic of < 0.05 was considered statistically significant. The percentage of the variability (I^2^) values of ≥ 25%, 50%, and 75% corresponded to the cut-off points for low, moderate, and high degrees of heterogeneity, respectively.

## Results

### Data selection

**Fig. 2:**
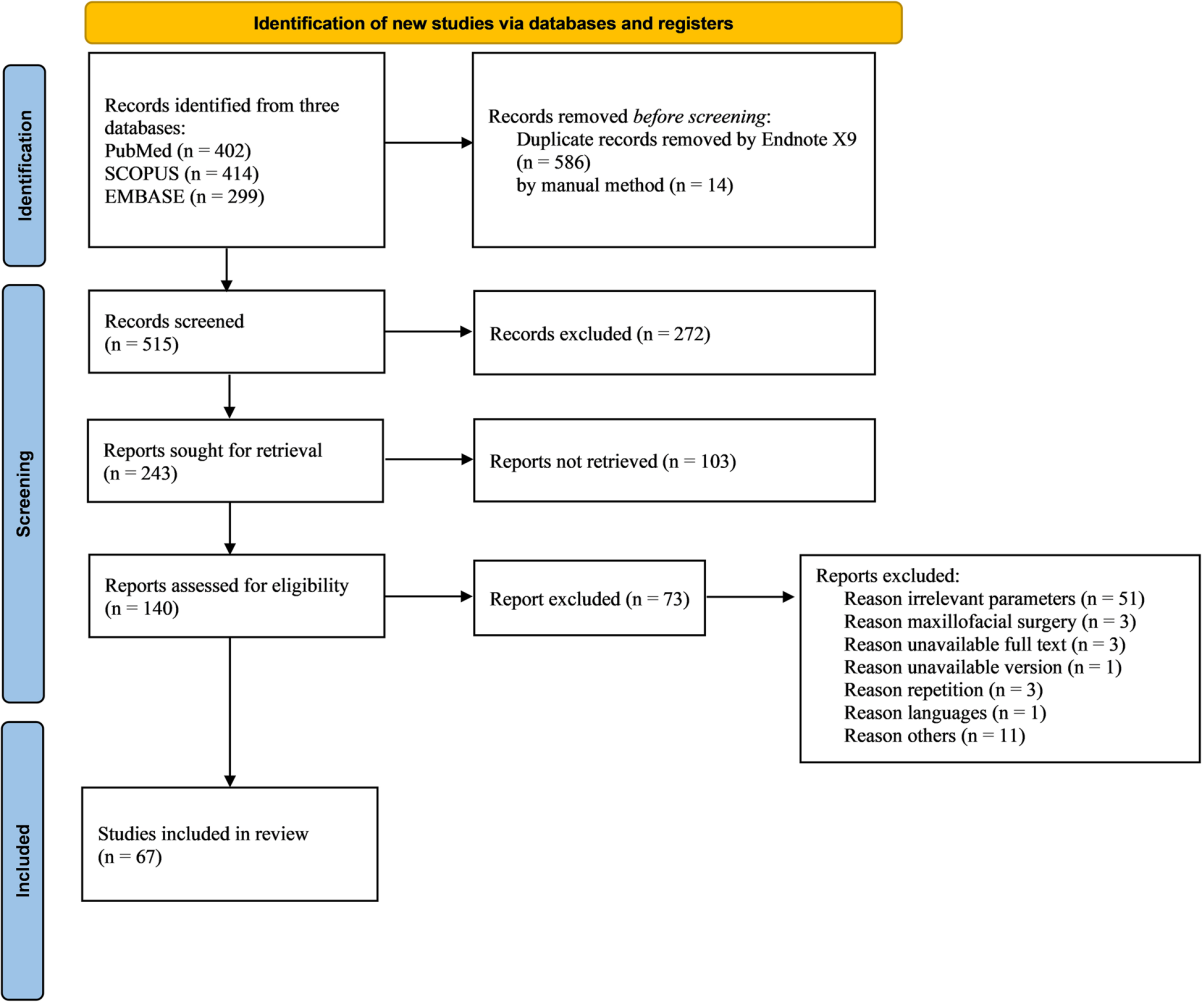
Modified PRISMA 2020 flow diagram of the process and results of literature search

According to the modified PRISMA 2020 diagram (Fig. 2), the initial electronic and manual search for studies through PubMed, Scopus, and Embase identified 1,115 articles. After the exclusion of duplicated articles, 515 articles were available for screening. Three hundred and seventy-five references were excluded after title and abstract screening. One hundred and forty lists were appraised for independent full-text reviewers. The inclusion and exclusion criteria were applied, and sixty-seven articles met the inclusion criteria and qualitative synthesis. The studies selected from the inclusion criteria are summarized in Table 5, 6, 7 and 8.

### Study characteristics

A total of 67 studies were included in this study. Of the 67 studies, 19 reported randomized clinical studies, 23 reported prospective studies, 21 reported retrospective studies, and 4 reported case studies. According to navigation systems (5,673 implants), 53 studies (4,504 implants) assessed the outcomes of static guided surgery, 15 studies (1,125 implants) reviewed the outcomes of dynamic guided surgery, and 2 studies (44 implants) analyzed the outcomes of robot-assisted surgery.

Comparisons between pre- and post- dental implant placements can be found among the three navigation types, namely, 43 studies on static systems [8, 18, 19, 21, 22, 30–67], 7 studies on dynamic systems [7, 16, 25, 68–71], and only 2 on robot-assisted surgery [11, 13]. Nevertheless, 15 studies [12, 15, 26, 72–83] showed comparative data between systems. To be more precise, seven studies assessed the accuracy between freehand and static navigation, three studies compared freehand and dynamic navigation, three studies compared static and dynamic systems, and two studies reported the difference between freehand, static, and dynamic systems.

### Quality of evidence

**Fig. 3:**
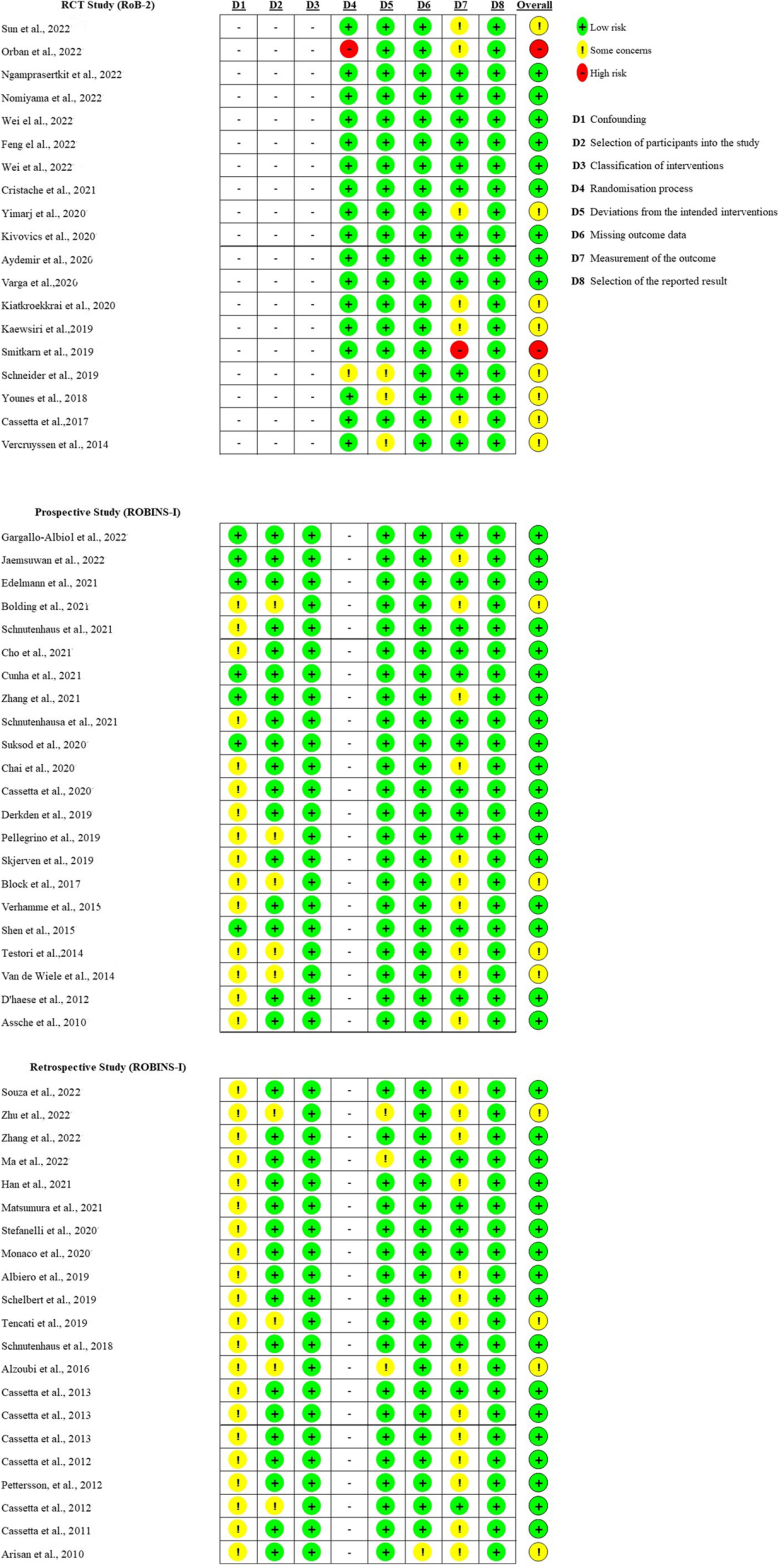
Summary of the bias risk assessment of the randomized controlled trial, prospective, and retrospective studies

**Fig. 4:**
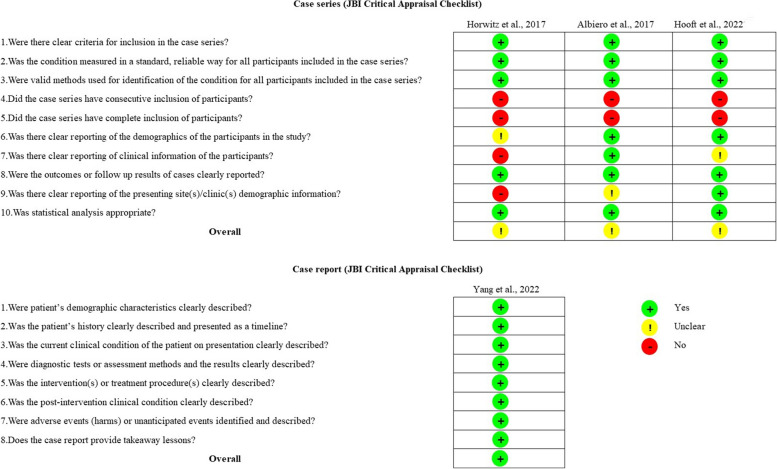
Summary of the bias risk assessment of the case studies

The bias risk analysis for the clinical studies that we included is summarized in Fig. 3 and Fig. 4. Overall, the nineteen RCTs [12, 22, 26, 42, 54, 59, 64–66, 69, 72, 74–77, 79–82] were assessed using RoB-2. Nine presented a “low risk” of bias, while eight presented “some concerns”. Two studies were identified as having a “high risk” of bias. Six RCTs showed “some concerns” regarding the outcome measurements because of their article’s awareness of outcome assessors. Meanwhile, one study was of “high risk” of bias. For “deviations from the intended interventions”, three studies exhibited “some concern”. Additionally, one study was of “some concern” and one study was of “high risk” in the randomization process.

Four articles showed “some concern” among selected prospective clinical studies [8, 11, 15, 16, 18, 21, 30, 38–40, 46, 49, 50, 52, 53, 55–58, 61, 68, 73, 83]. Overall, the eighteen articles appeared to be high-quality assessments. For the “confounding” factor, only eight articles were associated with a “low risk”, while fifteen were found to raise “some concerns” due to being uncontrolled for the critical or time-varying confounding domains. For the “measurement of the outcome” risk category, ten studies were unclear about the blinded assessor or used a non-blinded examiner. Meanwhile, twelve studies were classified as having a “low risk” of bias. Notably, “selection of participants into the study” was related to intervention and outcome, which was called selection bias. There were only five studies that exhibited “some concerns”. Additionally, there were classifications of “low risk” for all studies in “classification of the interventions”, “deviation from the intended interventions”, “missing outcome data”, and “selection of the reported result”.

Similar to the prospective study design, a total of twenty-one retrospective non-RCTs [7, 19, 25, 31–37, 41, 45, 47, 48, 51, 60, 62, 63, 67, 70, 78] were assessed using Robin-I. According to Fig. 3, concerns about “confounding” bias were referred to in all studies. For the outcome measurement, fourteen presented “some concerns”, while seven were classified as having a “low risk” of bias. Additionally, four studies were found to have “some concerns” when selecting participants. Similarly, there were three concerns in “deviations from intended interventions” and only one concern in “missing outcome data”. Overall, seventeen retrospective studies were “low risk”, while four were identified as showing “some concerns”.

According to Fig. 4, three case series [43, 44, 71] and one case report [13] were assessed using JBI Critical Appraisal Checklist. Conclusively, all case series were classified as having an “unclear” assessment, while only one case report seemed to be a high-quality assessment.

### Accuracy outcomes

#### Overall computerized guided implant placement

**Fig. 5:**
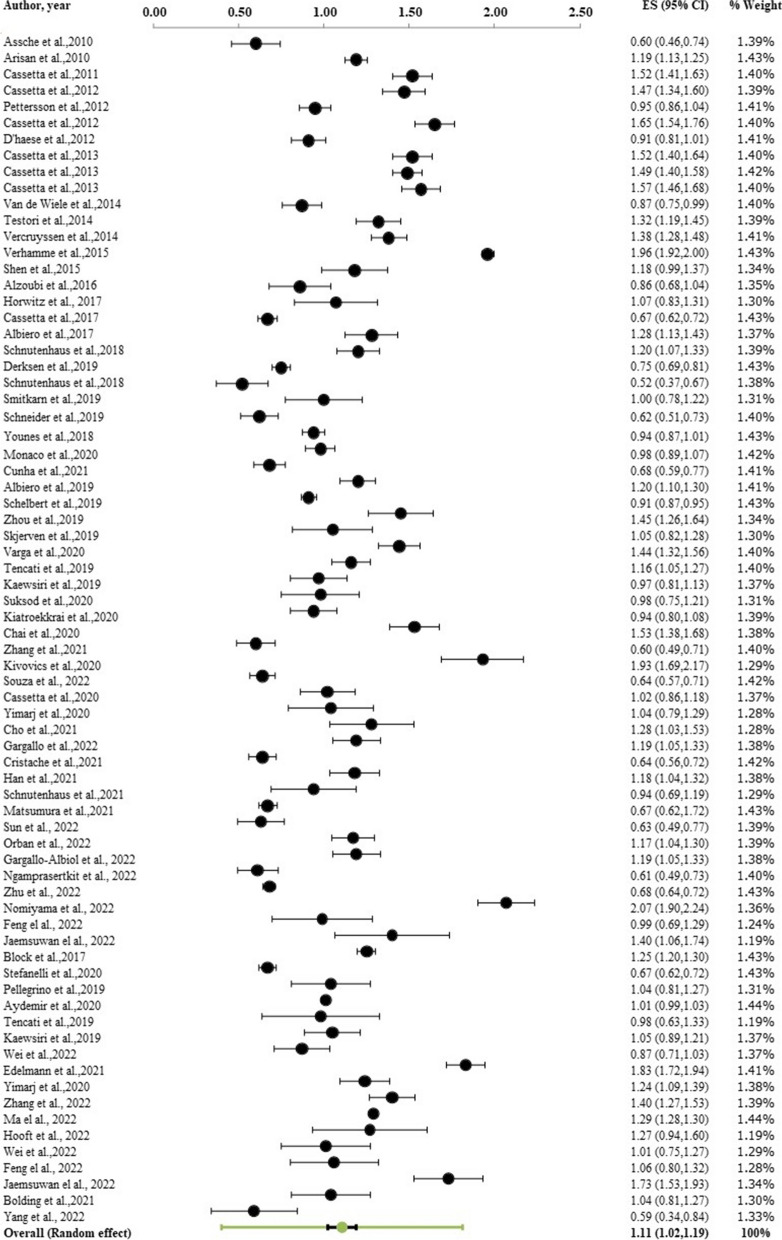
Forest plot showing global deviation (coronal) of computerized guided implant placement (95% CI)

**Fig. 6:**
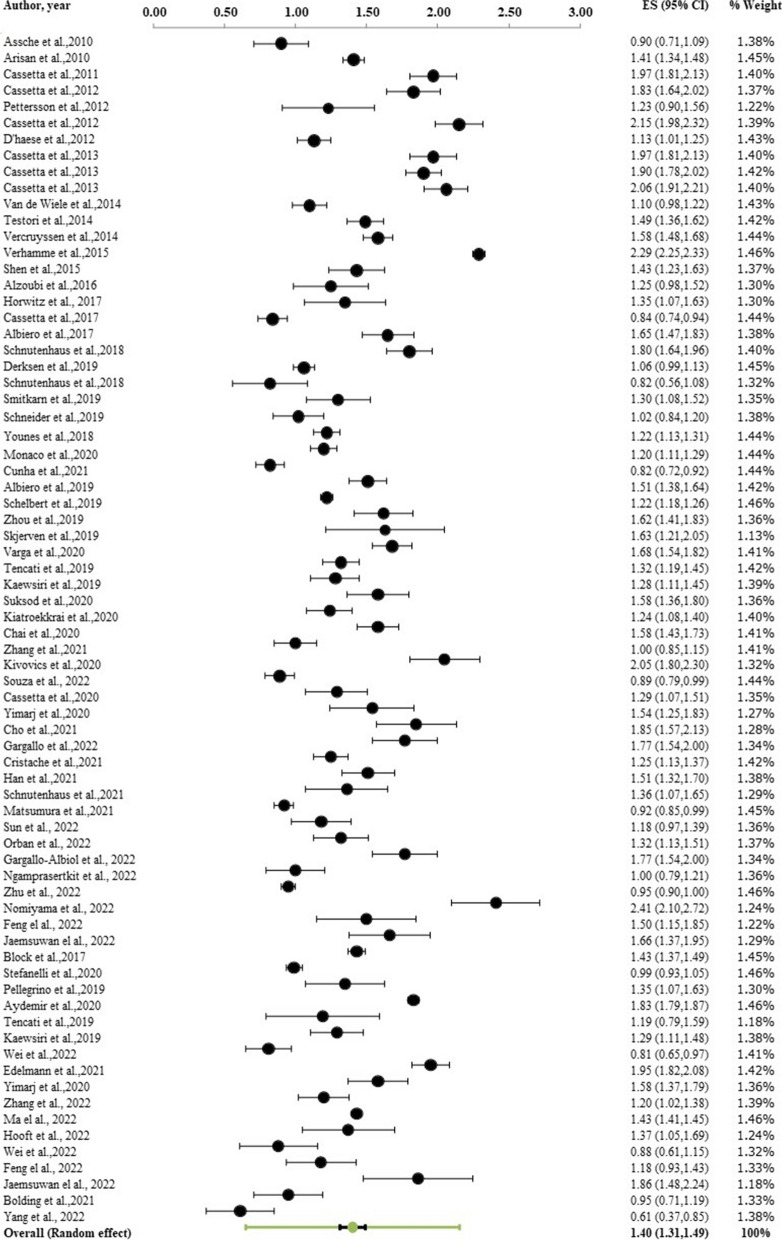
Forest plot showing global deviation (apical) of computerized guided implant placement (95% CI)

**Fig. 7:**
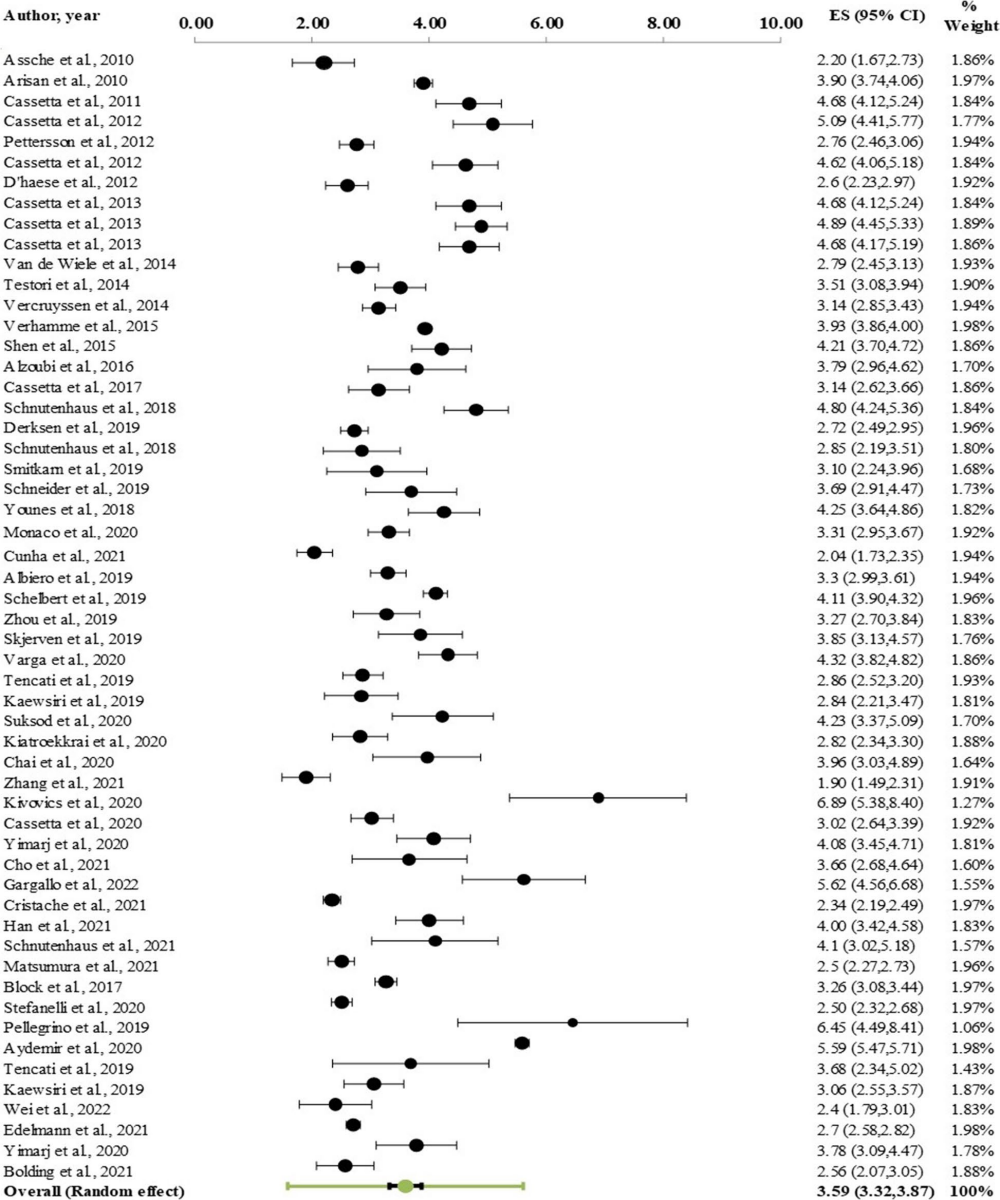
Forest plot showing angular deviation of computerized guided implant placement (95% CI)

Regarding global deviation at both the coronal and apical sections, and angular deviation in the implants, 67 studies (n = 5,673 implants) were divided into 53 static, 15 dynamic, and 2 robot-assisted surgeries. At the level of the implant shoulder, the overall weighted mean coronal deviation was 1.11 mm (95% CI: 1.02–1.19 mm; Fig. 5). At the level of the implant apex, the weighted mean apical deviation was 1.40 mm (95% CI: 1.31–1.49; Fig. 6). The overall weighted mean angular deviation was 3.51˚ with a 95% CI of 3.27˚-3.75˚ (Fig. 7).

#### Subgroup analysis: navigation system

**Fig. 8:**
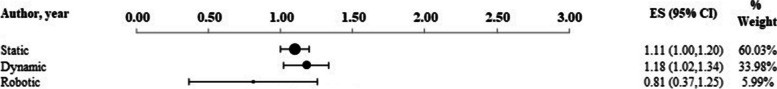
Forest plot showing global deviation (coronal) of static-, dynamic-, and robotic-guided surgery (95% CI)

**Fig. 9:**
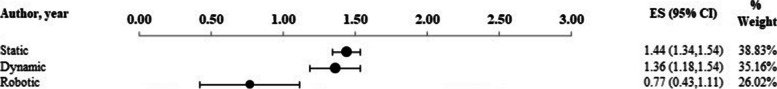
Forest plot showing global deviation (apical) of static-, dynamic-, and robotic-guided surgery (95% CI)

**Fig. 10:**
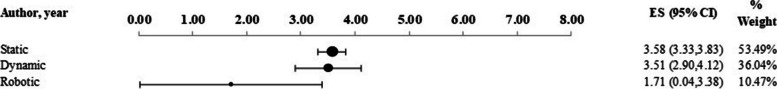
Forest plot showing angular deviation of static-, dynamic-, and robotic-guided surgery (95% CI)

The static, dynamic, and robotic approaches displayed a mean deviation at the entry point of 1.11 mm (95% CI: 1.00–1.20), 1.18 (95% CI: 1.02–1.34), and 0.81 (95% CI: 0.37–1.25), respectively (Fig. 8). At the apical point, the overall weighted mean deviation was 1.44 mm (95% CI: 1.34–1.54) in the static system, 1.36 (95% CI: 1.18–1.54) in the dynamic system, and 0.77 (95% CI: 0.43–1.11) in robot-assisted surgery (Fig. 9). For the angular deviation, the overall weighted mean deviation of these systems was 3.58˚ (95% CI: 3.33–3.83), 3.51˚ (95% CI: 2.90–4.12), and 1.71˚ (95% CI: 0.04–3.38), respectively (Fig. 10).

#### Effect of the arch type on the dynamic navigation system (maxillary or mandibular arch)

**Fig. 11:**
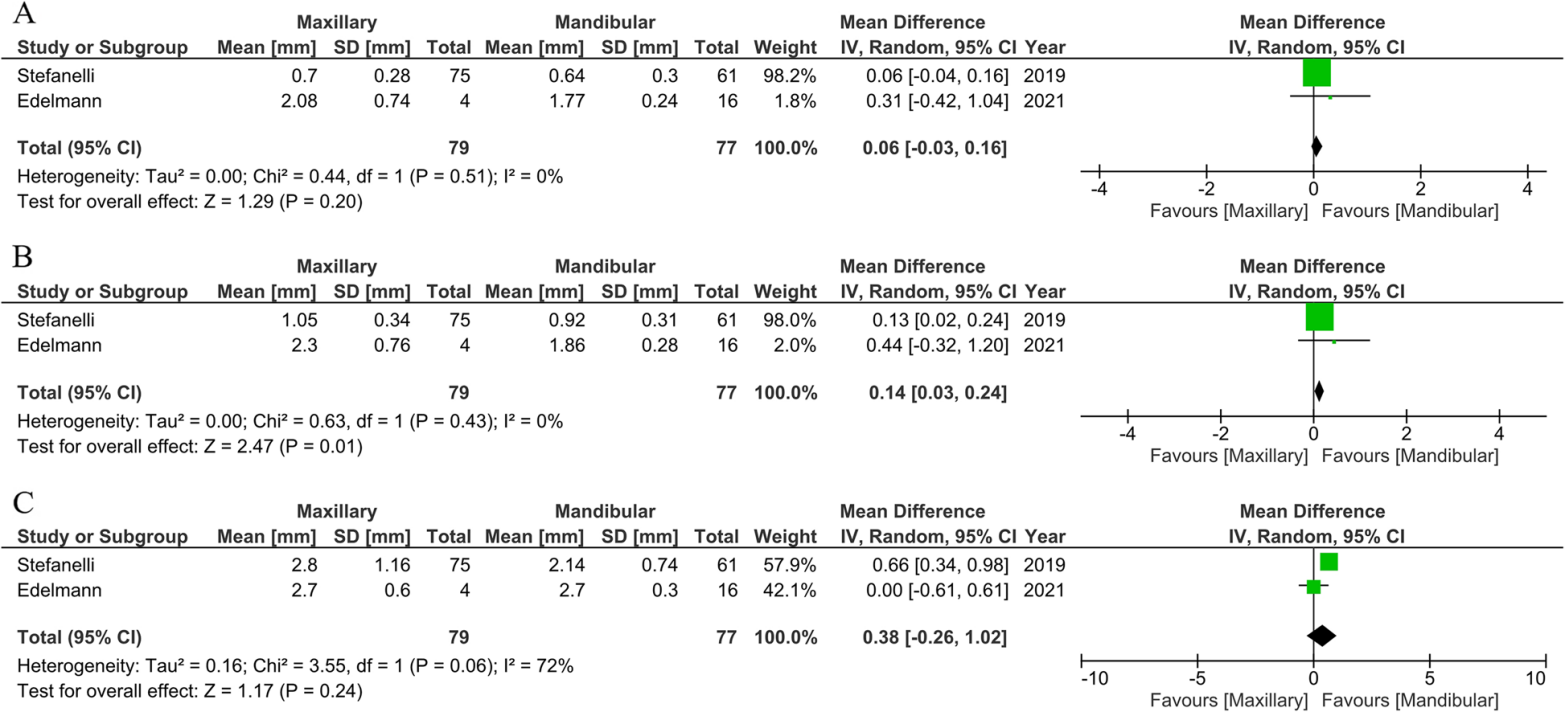
Forest plots of global coronal (**A**), global apical (**B**), and angular deviation (**C**) comparing clinical study of dynamic system in different arches

One prospective [68] and one retrospective study [25] (*n* = 156 implants) were reviewed to compare the accuracy of dynamic navigation surgery performed on the maxillary or mandibular arch. The meta-analysis data showed no statistically significant differences in global coronal deviation (MD:0.06 mm; 95% CI: -0.03 to 0.16; *P* ≥ 0.001; I^2^ = 0%), global apical deviation (MD:0.14 mm; 95% CI: 0.03 to 0.24; *P* ≥ 0.001; I^2^ = 0%), or angular deviation (MD:0.38 mm; 95% CI: -0.26 to 1.02; *P* ≥ 0.001; I^2^ = 72%) when comparing the maxillary and mandibular arches. Coronal and apical deviations exhibited a low degree of homogeneity between the studies. In comparison, angular deviation showed a high degree of heterogeneity between the studies (Fig. 11).

#### Effect of the flap operation on the dynamic navigation system (flapless and open-flap)

**Fig. 12:**
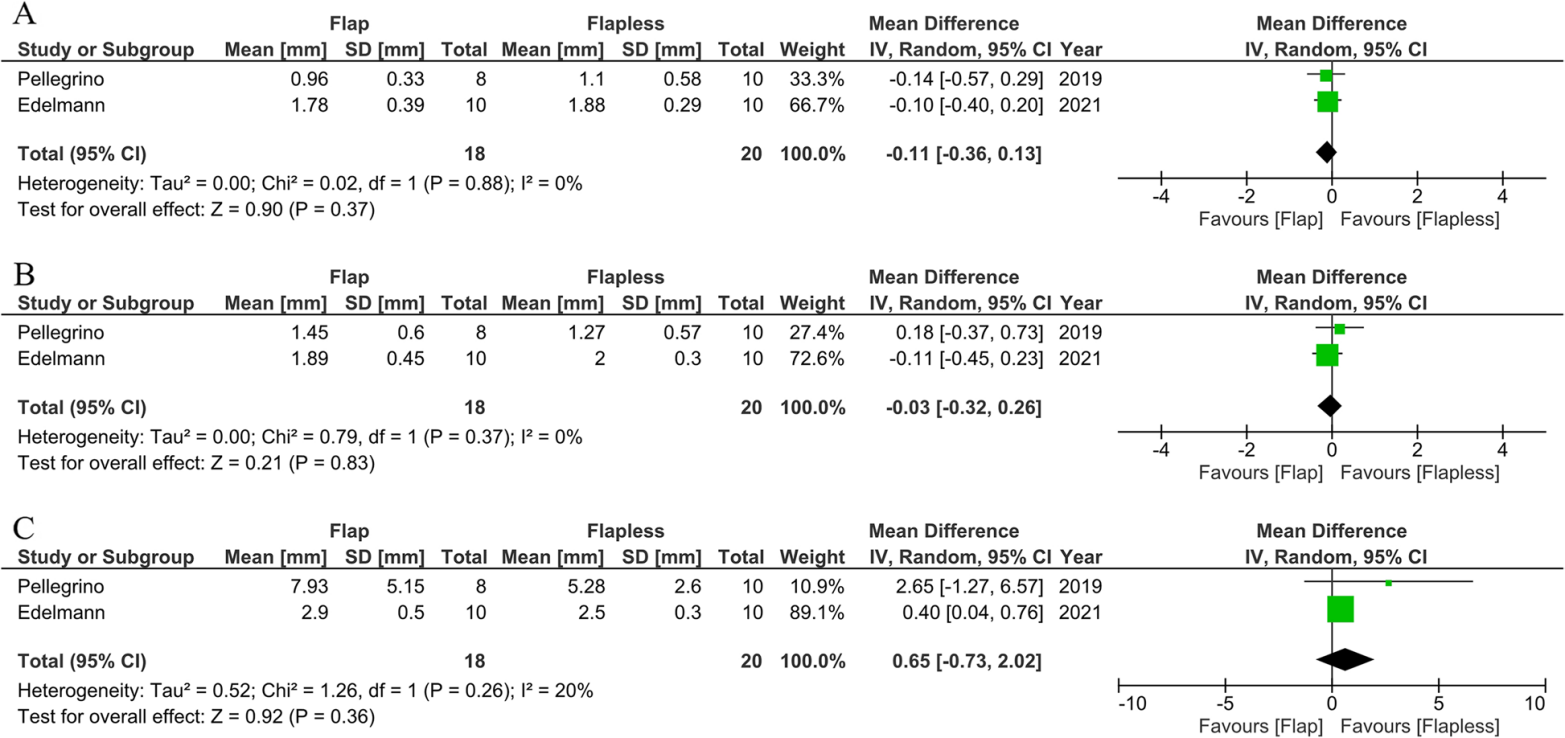
Forest plots of global coronal (**A**), global apical (**B**), and angular deviation (**C**) comparing clinical study of the dynamic system between the flap and flapless protocols

Only two articles [16, 68] (prospective studies; *n* = 38) were evaluated to compare the accuracy of dynamic navigation surgery performed using flapless and open-flap procedures. Two studies reporting data in a meta-analysis described no statistically significant differences in global coronal deviation (MD:-0.11 mm; 95% CI: -0.36 to 0.13; *P* ≥ 0.001; I^2^ = 0%), global apical deviation (MD:-0.03 mm; 95% CI: -0.32 to 0.26; *P* ≥ 0.001; I^2^ = 0%), and angular deviation (MD:0.65 mm; 95% CI: -0.73 to 2.02; *P* ≥ 0.001; I^2^ = 20%) (Fig. 12). No heterogeneity was observed between the studies on coronal and apical deviations. A low degree of homogeneity between the studies was found in the angular deviation.

#### Effect of the surgical protocol for a static navigation system (pilot, partial, or full protocol)

**Fig. 13:**
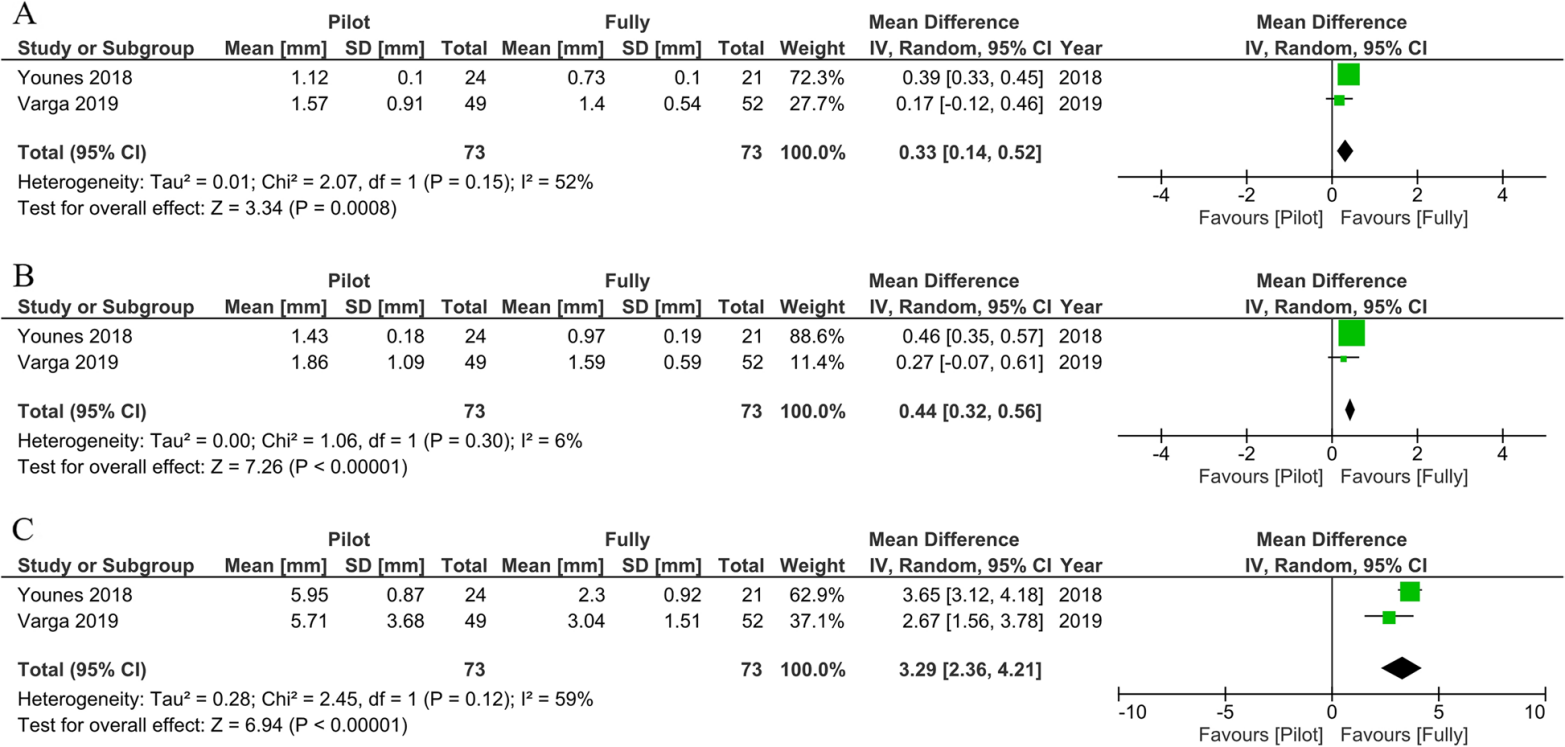
Forest plots of global coronal (**A**), global apical (**B**), and angular deviation (**C**) comparing clinical study of the static system between pilot and fully protocols

Two RCT studies [76, 77] (*n* = 146 implants) were reported to compare the accuracy of static navigation systems performed using pilot and fully guided protocols. The MD meta-analysis reported statistically significant differences favoring the full protocol in global coronal deviation (MD:0.33 mm; 95% CI: 0.14 to 0.52; *P* < 0.001; I^2^ = 52%), global apical deviation (MD:0.44 mm; 95% CI: 0.32 to 0.56; *P* < 0.001; I^2^ = 6%), and angular deviation (MD:3.29 mm; 95% CI: 2.36 to 4.21; *P* < 0.001; I^2^ = 59%). A medium-to-high heterogeneity between the studies was found for coronal and angular deviations. However, a low degree of homogeneity was found for apical deviation (Fig. 13).

**Fig. 14:**
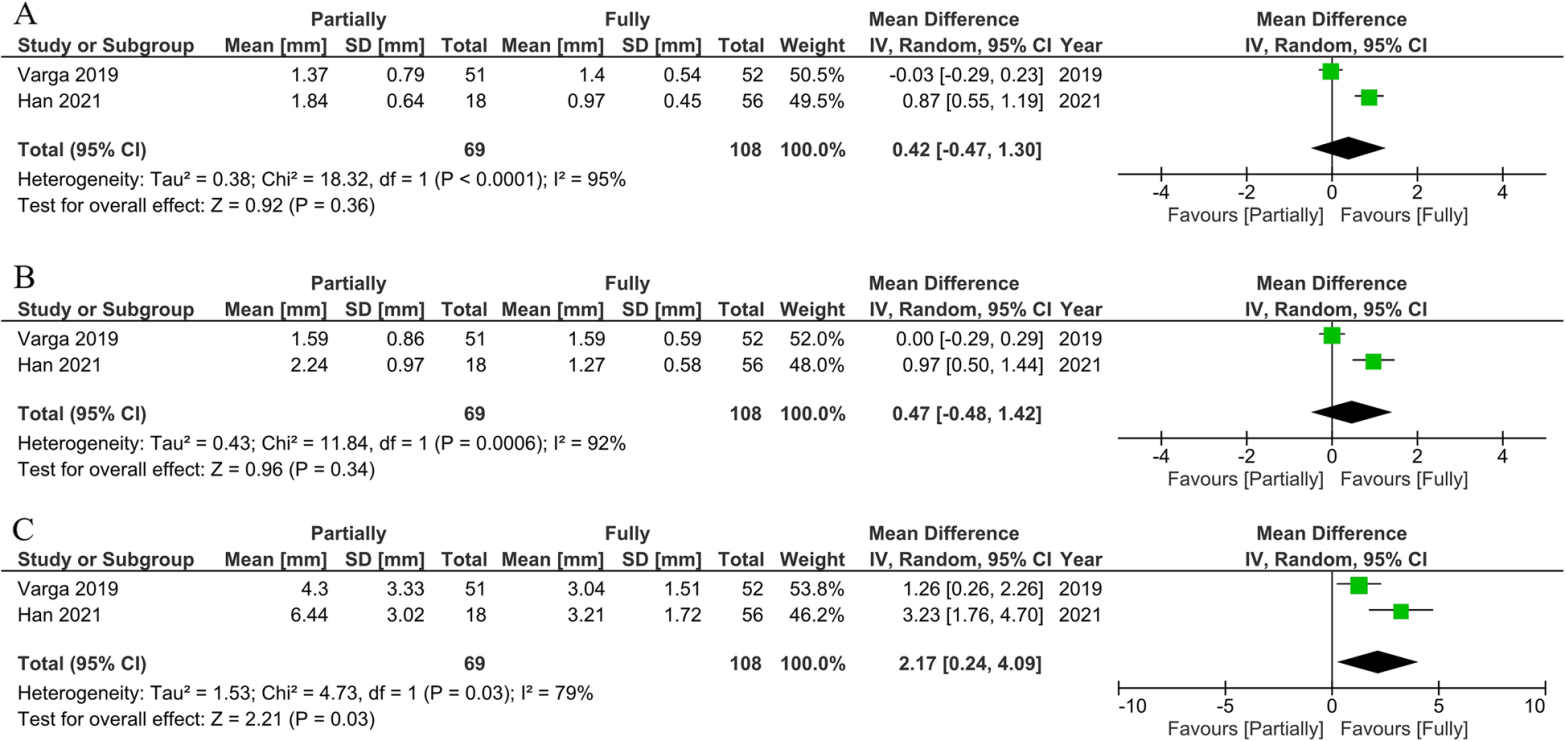
Forest plots of global coronal (**A**), global apical (**B**), and angular deviation (**C**) comparing clinical study of static system between partial and fully protocols

On the other hand, one RCT [77] and one retrospective study [60] (*n* = 177 implants) found no statistically significant difference between the partial and full protocols in global coronal deviation (MD:0.42 mm; 95% CI: -0.47 to 1.30; *P* = 0.39; I^2^ = 95%), global apical deviation (MD:0.47 mm; 95% CI: -0.48 to 1.42; *P* = 0.34; I^2^ = 92%), and angular deviation. (MD:2.17 mm; 95% CI: 0.24 to 4.09;*P* = 0.03; I^2^ = 79%). A high degree of heterogeneity between the studies was found for angular, coronal, and apical deviations (Fig. 14).

## Discussion

This systematic review evaluated the clinical accuracy of computer-aided static, dynamic, and robot-assisted surgery in implant surgery, along with the related factors. The result showed sixty-eight clinical studies analyzing the accuracy of computerized-implant surgery and determined that the average global coronal deviation, global apical deviation, and angular deviation were 1.11 mm, 1.40 mm, and 3.51˚, respectively. In comparison to the word by Jorba-Garcia et al. [10], these findings demonstrated a comparable discrepancy in the overall coronal and apical parts, albeit with a slightly lesser angular deviation. This may be due to the wider scope of our systematic review, which included articles specifically focused on robotic CAIS.

Our included studies showed two clinical reports [11, 13] assessing the accuracy of robot-assisted implant systems. These reports indicated that this novelty outperformed static and dynamic systems in terms of accuracy and precision. To date, an autonomous robotic system has achieved better 3D accuracy than the Yomi system. However, it should be noted that an autonomous robotic system performing under a maxillary tooth-supported guide may provide a more stable robotic splint base. On the other hand, the Yomi system used both maxillary and mandibular arches, with mucosa-supported guides. Generally, the robot-assisted system still presents some drawbacks, including the inability to operate on patients with limited mouth opening, and cannot be performed by inexperienced surgeons. Due to the lack of comparative cohort studies, it is crucial to take into account that the available data on this technology must be further explored with additional clinical proof.

Regarding the patients’ clinical conditions, our meta-analysis showed no statistically significant differences in coronal, apical, and angular deviation when comparing the maxillary and mandibular arches in dynamic systems. Two studies reported no difference in accuracy when inserting the implant between the upper and lower jaw [25, 68]. Nevertheless, there was a slight difference favoring the mandibular osteotomy. In contrast, a meta-analysis reported statistically significant differences in angular deviation favoring the mandibular arch in the case of static systems [20], explaining that the mandibular architecture is straighter than the maxillary arch, causing difficulties in controlling the angle of the dental bur. Another reason might be that the dense bone in the mandible could aid in restricting guided drilling and implant insertion.

Moreover, a higher bone density facilitates the segmentation step of the CBCT dataset and the registration procedure due to the high contrast of the images [60]. Stefanelli and colleagues noted that dynamics surgery easily provides direct access to the mandibular and maxillary operation sites. Meanwhile, static surgery performed with a thermoplastic stent [25] might alleviate the potential error in the maxilla and support our report that there is no difference between the maxilla and mandible. However, the available data in our review are relatively scarce, with few patients. Consequently, more data are needed for further evaluation and research.

Dynamic navigation can improve on computer-guided systems by adjusting the preoperative plan or evaluating the indiscernible vital structures underneath bone in real-time. Therefore, clinicians can perform the flapless protocol regardless of the limited visualization of the implant site. According to Zhou and colleagues [20], who compared the accuracy of static guided surgery between open-flap or flapless operations, the results showed a significant difference favoring the flapless procedure in terms of the accuracy of implant placement. This result is explained by the instability of a surgical guide when the flap is expanded. In addition to its superior accuracy, flapless surgery may reduce postoperative morbidities such as bleeding and patient discomfort, as well as time consumption [89]. Our meta-analysis compared open-flap and flapless surgery in the case of dynamic navigation. Dissimilar to the summarized review for guided surgery, there was no significant difference in angular, coronal, and apical deviation between the two methods. Two prospective studies [16, 68] showed no significant differences between the groups with no surgical template in the operation area, and the reflected tissue may not be interfered with by the guide. Although flapless surgery is more favorable than open-flap surgery, the operator should cautiously consider the preoperative procedure before launch. In case of an insufficient keratinized mucosa (less than 2 mm), a complete lack of keratinized tissue occurred at the buccal aspect after prosthetic restoration. Therefore, flapless surgery must only be performed if sufficient keratinized tissue is available [21]. Due to few patients being included in our meta-analysis (*n* = 38), further studies must be conducted to allow a conclusive summary.

Different guided surgical protocols were analyzed in our study. The implant protocols are typically categorized into three main types: pilot, partial or half, and complete (full) protocols. The fully-guided protocol, also known as the complete protocol, has been evaluated for its accuracy in various clinical studies. This protocol aids the operator at each surgical step, from the initial osteotomy to the insertion of the implant. Meanwhile, a guided template is only used in the osteotomy, but the operator still installs an implant free handed, which is classified as a partial (half) protocol. A pilot protocol is then used when a clinician wants to initially locate an implant position before drilling and inserting via mental navigation. This meta-analysis review analyzed the accuracy of implant placement using static guided surgery by comparing the three protocols. Our study found significant differences favoring the fully guided protocol in all directions. Other meta-analyses also illustrated the greater accuracy of the full protocol [89, 90]. Younes and coworkers explained that significant deviations from virtual planning might occur in every drill sequence from the pilot drill, so the clinician should passively insert the implant to escape these cumulative errors. Another critical procedure that reduces depth deviation uses the stereolithographic template for fully guided surgery [76]. Interestingly, Varga et al. found statistically significant differences only in terms of angular deviation. Accurate angulation is most important in the case of screw-retained restoration or an angulated abutment because misangulation can detrimentally influence the type of prosthetic restoration [77].

On the contrary, our meta-analysis revealed no significant difference between partially and fully guided surgery. The clinical studies described a greater accuracy provided by the fully guided protocol than by the partial protocol.Varga and coworkers suggested that “the higher the level of guidance is, the higher the correspondence between the planned and the actual implant position”. The higher correspondence can imply that the fully and partially guided surgery shows a safer guided option in terms of accuracy than pilot-guided or freehand surgery [77]. On the other hand, a previous meta-analysis [20] showed a statistically significant greater deviation in all parameters when comparing partially and fully guided protocols. They authors described how insertion by hand instead of a guide led to a more significant error in partially guided surgery. Notably, Zhou and colleagues included former publications that were not the same as those in our review. Therefore, the different results may be caused by the technological improvements in surgical-guided system. Due to the cost-effectiveness of guided surgery and some limitations to this approach, partially guided surgery is acceptable and widely used in clinical applications [76].

Aside from statics navigations, dynamic navigation can be divided into two protocols: drilling holes and fully guided implant placement. The fully guided protocol uses a dynamic procedure to initially drill the first drill until complete implant installation. Partial guidance refers to an operator performing osteotomy using the dynamic system and the implant seating of at least half of its length by hand [15]. A previous meta-analysis [9] reported no statistically significant differences between the partially and fully guided protocols, finding a slight difference favoring fully guided implantation because the fully guided placement proceeded at a slower speed than the drill-hole method. However, further studies should explore the clinical relevance of this comparison.

The 4th EAO consensus conference 2015 [91] stated the standardized postoperative follow-up of implant-related parameters: the 3D implant position, the peri-implant bone structures and morphology, the mucosa color, contour, and color of the reconstruction, and the extent of the restorative misfit. Moreover, the latest EAO [92] recommends using patient-reported outcome measurements (PROMs) in all clinical research. PROMS included the oral health impact profile (OHIP), the standardized use of the visual analog scale (VAS) for pain and discomfort, cost–benefit analysis, the time efficiency factor, and complication rates. Nevertheless, most clinical research has been driven by the implant-reported outcomes, especially in accuracy, without focusing on PROMs, which should constitute the virtual endpoint of clinical trials.

CBCT is usually an effective tool for assessing the accuracy measurement in clinical research for evaluating hard tissue, soft tissue, and implant position in 3D features. Despite the advantages of CBCT over other measurements, the ALARA (as low as reasonably achievable) rule should be followed for patient safety [91]. Currently, the analysis of implant placement position can be carried out with the utilization of a model scanner, intra-oral scanner, and CBCT. Akira and colleagues [23] investigated the consistency of measurements and the degree of shrinkage across three different modalities. The findings indicated that the shrinkage in CBCT was the most significant among the three modalities, primarily due to factors inherent to the system. They also stated that the data matching between CBCT and scanner measurements necessitates careful consideration regarding the accuracy of the values obtained with these devices. Furthermore, Derksen and coworkers reported that postoperative intraoral scan and postoperative CBCT scan techniques had similar accuracy outcomes. They recommended that more studies be conducted to confirm this hypothesis [21].

The limitation of this systematic review and meta-analysis involves the heterogeneity among the included studies’ static, dynamic, and robotic systems, regardless of the different study designs. The potential factors in each system are the lists explained earlier that contribute to the cumulative errors. Apart from these, possible factors include guide support, guide stability, the restriction of access during surgery (location and limited mouth opening), the movement of the patient, the edentulous space, the time of placement, the characteristics of the implant (material, diameter, length), the operator’s experience, or different postoperative assessment, which may result in an undesirable outcome. Moreover, the quality of studies, including considering a prospective study and a case series, should be realized. Overall, the randomized controlled trials appeared to be of some concern, whereas non-randomized controlled trials were classified as high quality. A common consensus should be followed to avoid potential bias in applying this study’s results.

## Conclusions

Within the limitations of our reviews, the robotic system consistently exhibited the least amount of deviation, followed by the dynamic and static systems. Nevertheless, the increased accuracy achieved via robotic guided surgery should be taken with caution until further research and technology is available. Considering the relevant factors, no significant differences were found between the arch and flap approaches in the dynamic systems. In the case of the static systems, there were statistically significant differences observed between the pilot and fully guided protocols, but no significant differences were found between the partially and fully guided protocols. Moreover, it is the consensus of several studies that a fully guided protocol is the gold standard in clinical practice. Future clinical research should focus on exploring the application of robot-guided systems in clinical settings to enhance the accuracy of implant placement and PROMs utilizing implant-assisted systems.

## Data Availability

All data generated and analyzed for the review are available upon request from the authors (Corresponding author: pimduen.rungsiyakull@cmu.ac.th).

## Abbreviations

CBCT: Cone-beam computed tomography (CBCT)
sCAIS: Static computer-aided implant surgery
dCAIS: Dynamic computer-aided implant surgery
DICOM: Digital imaging and communications in medicine
STL: Standard Template Library
